# Why do valence asymmetries emerge in value learning? A reinforcement learning account

**DOI:** 10.3758/s13415-022-01050-8

**Published:** 2022-12-28

**Authors:** Chenxu Hao, Lilian E. Cabrera-Haro, Ziyong Lin, Patricia A. Reuter-Lorenz, Richard L. Lewis

**Affiliations:** 1 Department of Psychology, University of Michigan, Ann Arbor, MI USA; 2 Chair of Autonomous Systems and Mechatronics, Department of Electrical Engineering, Friedrich-Alexander Universität Erlangen-Nürnberg, Erlangen, Germany; 3 Amazon, Berlin, Germany; 4 Weinberg Institute for Cognitive Science, University of Michigan, Ann Arbor, MI USA

**Keywords:** Reinforcement learning, Learning asymmetries, Value-based decisions

## Abstract

The Value Learning Task (VLT; e.g., Raymond & O’Brien, 2009) is widely used to investigate how acquired value impacts how we perceive and process stimuli. The task consists of a series of trials in which participants attempt to maximize accumulated winnings as they make choices from a pair of presented images associated with probabilistic win, loss, or no-change outcomes. The probabilities and outcomes are initially unknown to the participant and thus the task involves decision making and learning under uncertainty. Despite the symmetric outcome structure for win and loss pairs, people learn win associations better than loss associations (Lin, Cabrera-Haro, & Reuter-Lorenz, 2020). This learning asymmetry could lead to differences when the stimuli are probed in subsequent tasks, compromising inferences about how acquired value affects downstream processing. We investigate the nature of the asymmetry using a standard error-driven reinforcement learning model with a softmax choice rule. Despite having no special role for valence, the model yields the learning asymmetry observed in human behavior, whether the model parameters are set to maximize empirical fit, or task payoff. The asymmetry arises from an interaction between a neutral initial value estimate and a choice policy that exploits while exploring, leading to more poorly discriminated value estimates for loss stimuli. We also show how differences in estimated individual learning rates help to explain individual differences in the observed win-loss asymmetries, and how the final value estimates produced by the model provide a simple account of a post-learning explicit value categorization task.

**Supplementary Information:**

The online version contains supplementary material available at 10.3758/s13415-022-01050-8.

## Introduction

Most real world decision making involves uncertainty. For example, the decider may be uncertain about the value of different options, the likelihood of payoffs from different choices, or both. An important class of such decisions are *value-based decisions*, where one processes the information about decision options, estimates their values, and makes a choice based on the value estimates (Rangel, Camerer, & Montague, 2008).

In this work, we build on two related definitions of *value*. The first is the economic value of an option (Brosch & Sander, 2013), usually defined as expected value or expected subjective utility—the value or utility of an outcome multiplied by its probability of occurring (Savage, 1972). A related and more general definition of value is specified in computational reinforcement learning (RL) theory (Sutton & Barto, 2018), where the value of a state-action pair is the expected cumulative discounted future reward after taking an action in the given state (Gershman & Daw, 2017; Sutton & Barto, 2018). In the case of single-shot (i.e., non-sequential) decisions in which a choice only affects an immediate outcome, the two definitions of value are equivalent.

Values can be learned through experience (Kahneman, 2003; Gershman & Daw, 2017). This learning can be modeled within the RL framework, which provides functional accounts of the processes of value-based learning and choice with a formal computational theory, and has been used to provide theoretical foundations for understanding the underlying neural mechanisms of value learning (Montague, Hyman, & Cohen, 2004; Daw, Niv, & Dayan, 2005; Brosch & Sander, 2013). A key problem faced by any RL agent in an uncertain environment is balancing *exploration* in service of learning, and *exploitation* of learned value in service of obtaining reward (Sutton & Barto, 2018).

To understand how acquired value influences behavior, laboratory tasks have been developed to establish associations between otherwise neutral items and win or loss outcomes. The impact of value on subsequent processing has been examined in a variety of cognitive processing domains such as attention (Della & Chelazzi, 2009; Raymond & O’Brien, 2009), motor control (Painter, Kritikos, & Raymond, 2014), and memory (Aberg, Müller, & Schwartz, 2017). We focus here on a task which we refer to as the *Value Learning Task* (VLT; Raymond & O’Brien, 2009). The VLT has been used to examine how value learned through trial-by-trial experience impacts the cognitive processing (e.g. visual attention, perceptual and motor processing) of stimuli that were previously associated with wins or losses that occurred with low or high probability. This paradigm has also been used to investigate learning within win and loss contexts (Palminteri, Khamassi, Joffily, & Coricelli, 2015; Palminteri & Lebreton, 2021).

Research adopting the VLT has largely focused on examining the cognitive processing of stimuli previously associated with wins or losses, and not the learning itself or possible valence asymmetries in the learning. But a recent meta-analysis of several VLT experiments (Lin et al., 2020) provides evidence that people learn win associations better than loss associations. Furthermore, in two new empirical studies, Lin et al., (2020) demonstrated that this learning asymmetry was evident with both monetary earnings and non-monetary points, and was evident regardless of whether participants received explicit instructions about the outcome contingencies.

This learning asymmetry has been referred to as the *punishment learning paradox* in past work (Mowrer, 1960; Moutoussis, Bentall, Williams, & Dayan, 2008; Maia, 2010; Palminteri et al., 2015; Palminteri & Lebreton, 2021), and Palminteri et al., (2015) provide an intuitive explanation from the perspective of learning within a loss context; we discuss this explanation below and its relation to our model.

In the present computational study, we provide a clear explanation of the observed asymmetries based on RL computational theory (Sutton and Barto, 2018). Crucially, our RL model must grapple with the exploration-exploitation problem imposed by the initial uncertainty of the setting. We next describe the VLT and key empirical findings in more detail before introducing the computational learning model.

## The value learning task

The Value Learning Task (VLT) involves a choice game where a pair of images is presented on each trial, and participants select one image from each pair, receiving a probabilistic positive, negative, or zero reward as feedback. The participants’ goal is to maximize earnings (points or money) by learning and exploiting the expected value of each stimulus.

An example of the probabilistic structure of a typical VLT paradigm is given in Table 1. There are pairs of images in *win*, *loss*, or *no-change* conditions. In the *win* condition, a selection between a pair of images results in a win of 5 points 80*%* of time, and 0 points 20*%* of time; in the *loss* condition, a selection between a pair of images results in a loss of -5 points 80*%* of time and 0 points 20*%* of time; in the *no-change* condition, a selection between a pair of images always results in 0 points (Table 1).

**Table 1:** The standard symmetric payoff structure used in the VLT: the high probability win (A) and high probability loss (C) stimuli have the same absolute rewards and expected values, as do the low probability win (B) and low probability loss (D) stimuli

Condition	Stimulus	Outcomes and Probabilities	Expected Value
Win pair	A	+ 5(*p* = 0.8), 0(*p* = 0.2)	4
Win pair	B	0(*p* = 0.8), + 5(*p* = 0.2)	1
Loss pair	C	− 5(*p* = 0.8), 0(*p* = 0.2)	− 4
Loss pair	D	0(*p* = 0.8),− 5(*p* = 0.2)	− 1

The structure of the task is *symmetric* in that corresponding stimuli from each valence condition have the same absolute expected values, as a consequence of the symmetry of the probabilities and rewards. To maximize earnings, participants must learn to select the image associated with the highest expected value within each pair. In other words, the optimal choice for the win pair is the high probability win image (80% win), whereas the optimal choice for the loss pair is the low probability loss stimulus (20% loss).

The VLT has been adopted by many researchers to examine the impact of learned value on perceptual and attentional processing by presenting the VLT stimuli in a variety of secondary tasks where the reward schedule is discontinued and no longer task relevant. Despite numerous studies using the same VLT, the conclusions drawn from the secondary tasks have varied. For example, Raymond and O’Brien (2009) reported two effects of acquired value on old versus new recognition of faces when attentional capacity was limited. First, stimuli previously associated with high probability outcomes (either win or loss) showed a processing advantage (i.e. greater recognition accuracy) regardless of available attention (reduced versus full) compared to stimuli previously associated with low probability outcomes. Second, win-associated stimuli showed processing advantages (versus loss-associated stimuli) when available attention was reduced. In another example, a reach-to-grasp task showed faster reaches toward stimuli previously associated with high probability outcomes (versus low probability) but more efficient reaches toward stimuli previously associated with wins (versus loss or no-change; Painter et al., (2014)).

### Asymmetries in learning wins and losses

But inferences about asymmetric valence effects on subsequent processing depend, at least implicitly, on the assumption that the values of win and loss stimuli have been learned equally well—otherwise the subsequent processing differences may be due to learning differences rather than valence per se.

Lin et al., (2020) conducted a meta-analysis of studies adopting the VLT to compare learning for win and loss outcomes. In each study, the probabilistic structure was symmetric as in the example in Table 1 above. Nevertheless, the results of the meta-analysis showed that the probability of optimal choice was significantly higher for win-associated stimuli compared to loss-associated stimuli, suggesting a valence-based asymmetry. Furthermore, when Lin et al., (2020) conducted new experiments using the VLT, they found that the learning asymmetry was observed regardless of whether the outcome led to monetary or point earnings, and was also observed when participants were provided with a description of the task structure (with information about the specific probabilities and payoffs but not the association between stimuli and outcomes).

From the perspective of learning in the loss decision context, Palminteri et al., (2015) has provided an intuitive explanation: when the punishment, i.e., loss, is successfully avoided, the extrinsic negative reinforcement becomes infrequent due to avoidance and so the values of the negatively-valenced stimuli are learned less well. Our computational model shows exactly how and why this occurs in a learning agent grappling with the exploration-exploitation tradeoff.

### Asymmetries in explicit memory for wins and losses

One approach that Lin et al., (2020) have pursued to further understand the nature of the learning asymmetry is to probe participants’ explicit knowledge of the outcomes associated with each stimulus by using a post-learning memory task. In the studies conducted by Lin et al., (2020) participants completed a forced choice recognition memory task in which participants indicated the outcome most likely associated with each image from the VLT (e.g., “very likely to win” for the 80% win scene). Performance on the post-learning memory task was consistent with the learning asymmetry in the VLT: memory accuracy was superior for optimal win scenes versus optimal loss scenes—though interestingly, participants were overall more accurate in identifying the outcome associated with loss-associated images. We return to this finding later in the paper.

## The computational reinforcement learning model

We apply computational reinforcement learning (RL) theory (Sutton and Barto, 2018) to build models of the VLT in order to provide new insights and possible explanations for the observed win-loss asymmetry. Our model is simple, but it yields interesting explanations of qualitative phenomena from the results of trial-level simulations, and it also provides some insights into the nature of individual differences and performance on the subsequent outcome memory task described above.

RL theory (Sutton & Barto, 2018) provides a formal definition of the problem of learning from experience and insights on how to act so as to maximize cumulative rewards. In the standard RL problem formulation, a decision-maker, or an *agent*, determines at each time step *t* what action, *a*_*t*_, to take at a given state, *s*_*t*_ (or observation), and at the next time step receives some reward *r*_*t*+ 1_ and transition to a new state or observation. The agent’s goal is to maximize the expected cumulative future rewards. For example, in the VLT, the actions that result in maximum total reward are those actions that select the high probability win scene in the win condition and the low probability loss scene in the loss condition.

The Value Learning Task is a special case of the general RL problem in that it does not involve *sequential decision making*; i.e., each choice affects only immediate reward and not future rewards. The win and loss pairs in the VLT are thus each equivalent to a *two-armed bandit task*. Despite their simplicity, bandit tasks are nevertheless interesting in RL theory and algorithm development because they are the minimal setting which imposes the challenge of learning value from probabilistic outcomes along with the need to balance exploration and exploitation.

Sutton and Barto (2018) provide a number of algorithms for solving bandit tasks, including sophisticated methods that approach optimal exploration strategies. We adopt here a simple incremental algorithm that learns expected values via an error-driven learning rule. The form of the rule is shared by many RL algorithms and theoretical approaches to human and animal learning.

We denote the estimated value of action *a* at trial *t* as *Q*_*t*_(*a*). In the VLT, the value of an action is its expected reward. For example, the value for the high probability win scene (80% win with a reward of 5) is 5 ∗ 0.8 + 0 ∗ 0.2 = 4. The error-driven update is:

1 $$  Q_{t+1}(a) = Q_{t}(a) + \alpha(r_{t} - Q_{t}(a)) $$

where *α* is the agent’s learning rate; *α* ∈ [0,1]. When *α* is 0, there is no learning, and when *α* is 1, the agent only takes into account the feedback from the previous trial.

At each trial, the agent makes a selection according to a *choice rule* that converts current action value estimates into choices while balancing exploration and exploitation. There are several common choice rules, including *greedy* (always choose the action with the highest estimated value) and *epsilon-greedy* (choose a random action with probability *𝜖* otherwise choose greedily). We adopt here another standard choice rule for balancing exploitation and exploration: the *softmax* rule. According to the softmax rule, at trial *t*, the probability of choosing an action *A* given the value estimates for action *A* and *B* is:

2 $$  P(\!A|Q_{t}(A), Q_{t}(B)) = \frac{\exp(\beta * Q_{t}(A))}{\exp(\beta * Q_{t}(A)) + \exp(\beta * Q_{t}(B))} $$

where *β* is the inverse temperature parameter, and larger *β* corresponds to greedier choices (e.g., Daw 2011). The computed probabilities thus define a multinomial distribution from which an action is sampled; actions with higher value estimates are sampled more frequently, but lower-valued actions always have a nonzero probability. Table 2 provides an example of how the model updates action values, converts the values into choice probabilities, and samples an action choice for several trials in the loss condition given a specific pair of parameters (*α* = 0.23, *β* = 2).

**Table 2:** Model simulation example of a sequence of trials at the start of the Loss pair condition with *α* = 0.23, *β* = 2

t	Condition	*P*(*C*)	*P*(*D*)	Model choice	Reward	*Q*(*C*)	*Q*(*D*)
0						0	0
1	Loss	0.5	0.5	C	− 5	− 1.15	0
2	Loss	0.09	0.91	D	0	− 1.15	0
3	Loss	0.09	0.91	D	− 5	− 1.15	− 1.15
4	Loss	0.5	0.5	D	0	− 1.15	− 0.886
5	Loss	0.37	0.63	D	0	− 1.15	− 0.68
⋮	⋮	⋮	⋮	⋮	⋮	⋮	⋮

The model chooses between stimulus C and stimulus D in the pair. Value estimates for both choices, denoted *Q*(*C*) and*Q*(*D*) start at 0. On each trial, the model converts value estimates for choices into choice probabilities *P*(*C*) and *P*(*D*), makes a selection by sampling a choice according to these probabilities, receives a reward, and updates its value estimates using the error-driven update rule

Adopting the softmax rule has the analytic advantage of directly giving a nonzero probability for each choice on each trial conditioned on the learners value estimates, which allows us to use maximum likelihood estimation to find the best fitting parameters to the data. The ability of RL models to make contact with human data at the individual trial level is a significant theoretical benefit of their use (Daw, 2011). In the following section we provide details on how we select model parameters and modeling learning and choice in the VLT at the trial level.

Error-driven learning rules with fixed learning rates such as the rule we adopt in Eq. 1 may be contrasted with the simple method of keeping a running average of experienced rewards as value estimates. Rules such as Eq. 1 are effectively computing a *weighted average* of experienced rewards, where more recent rewards are weighted more than rewards in the distant past. Such rules have the advantage that they allow the agent to adapt to non-stationary environments where the probabilistic payoffs may be changing over time. They also require an *initial value estimate*, which can be a locus of prior knowledge about the environment. In the absence of prior knowledge, common initial value estimates are zero, very small random values with mean zero, or random values with a small positive mean; positive initial values estimates build in an optimism that is one method for encouraging exploration (Sutton & Barto, 2018). For our model we fix the initial value estimate to be zero and explore its implications.

The model thus has two free quantitative parameters that correspond to learning rate (*α*) and the balance between exploration and exploitation (*β*). These parameters influence how *Q*_*t*_(*a*) is updated and how the agent makes the selection at each trial. In our simulations below we explore two methods for setting the parameters: maximizing empirical fit to human data, and maximizing reward in the task.

## Simulating the Value Learning Task

### Experiment structure.

We simulate first the VLT in Lin et al., (2020). This task has three pairs of stimuli: one pair in the win condition, one pair in the loss condition, and one pair in the no-change condition, with payoffs and probabilities as in Table 1. The task has 300 trials across 5 blocks: 100 win pair trials, 100 loss pair trials, and 100 no-change trials.

Over these 300 trials the model thus estimates six values: the win-correct option, the win-incorrect option, the loss-correct option, the loss-incorrect option, and the two no-change options. (We focus here only on the values for the win and loss pairs as no learning happens for the no-change pair.) All initial values were set to zero in our analyses. On each trial, the model makes a choice given the condition and the softmax choice rule (Eq. 2), receives a reward probabilistically according the parameters in task (Table 1), and updates the value of the corresponding choice according to the incremental update rule (Eq. 1).

Because the point schemes and monetary currencies vary arbitrarily across VLT experiments, and any such points or currencies must be transformed by humans into an internal reward signal (Singh, Lewis, Barto, & Sorg, 2010), we use a standardized reward (1 and − 1) in all of our subsequent analyses.

### Setting model parameters

We simulated the VLT with parameters set in two ways: in the *data-driven approach* we estimate *α*,*β* for each individual participant to maximize fit to their choice data (details below). In the *theory-driven* approach we find optimal settings of *α* and *β*—settings that maximize expected reward in the task. This represents a simple bounded optimality analysis to find *computationally rational* (Lewis, Howes, & Singh, 2014) parameter settings that determine the upper bound on performance given the constraints of the learning algorithm.

We use maximum likelihood estimation to find the pair of parameters that yields choices that best fit each human participant’s choices. The likelihood is given directly by the softmax rule (Daw, 2011), and the likelihood or probability of the entire observed sequence of choices from one participant is the product of the probabilities of their choices on all trials:

3 $$  \prod\limits_{t} P(c_{t} = A|Q_{t}(A), Q_{t}(B)). $$

The product in Eq. 3 is often an extremely small number and so it is usually better to compute the summed log-likelihood instead, which is

4 $$ \begin{array}{@{}rcl@{}} &&\sum\limits_{t} \log\left( P(c_{t} = A|Q_{t}(A), Q_{t}(B))\right)=\sum\limits_{t} \beta * Q_{t}(A) \\&&\quad- \sum\limits_{t} \log(\exp(\beta * Q_{t}(A))+ \exp(\beta * Q_{t}(B))). \end{array} $$

To find *α*,*β* that maximize the quantity in Eq. 4 we use a simple randomized grid search, sampling 100 *α*’s from a uniform distribution, $\mathcal {U}(0,1)$, and 100 *β*’s from a uniform distribution, $\mathcal {U}(0,10)$, which resulted in 10,000 pairs of parameter settings. For each pair we computed the log-likelihood for each individual participant’s data given each pair of parameters and the model. Finally, we chose the pair of parameters that produced the largest log-likelihood as the maximum likelihood estimation of each individual’s learning rate and selection strategy (Daw, 2011).

**Fig. 1:**
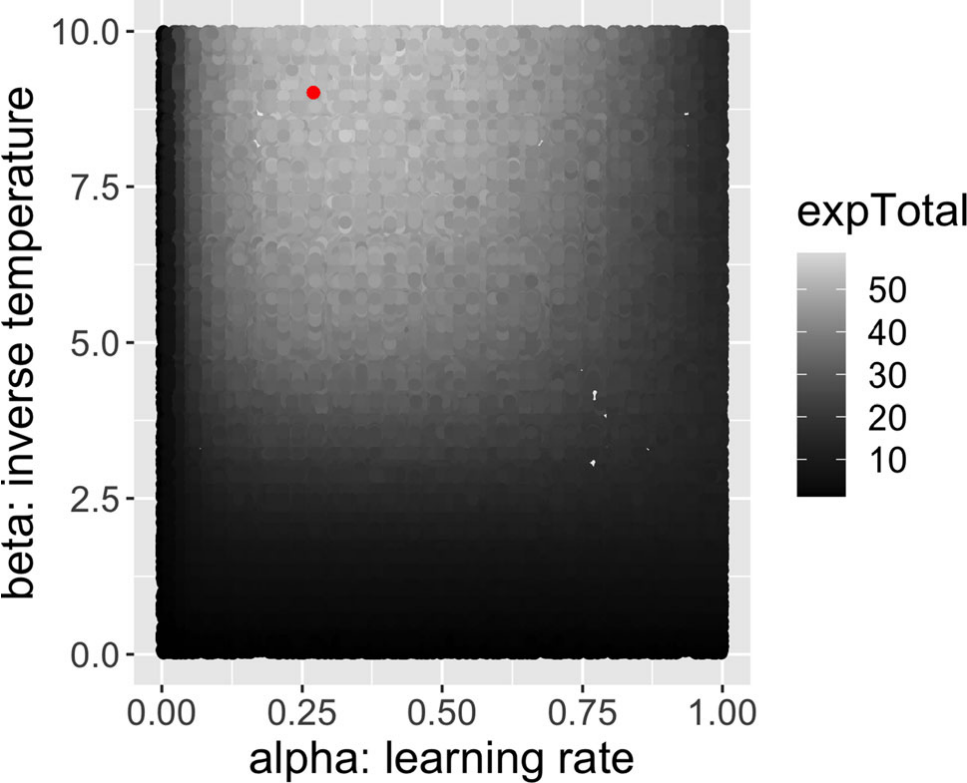
Total payoff given different values of *α* and *β*. The pair of parameters that produce the highest reward is *α*^∗^ = 0.27,*β*^∗^ = 9.02, shown as a red dot. We have applied an exponential transform (1.07^total reward+ 5^) to the simulated accumulated rewards to make the visualization clear

We found an approximation of the optimal pair of parameters for the task with the same randomized grid search: we sampled 100 *α*’s from a uniform distribution $\mathcal {U}(0,1)$, and 100 *β*’s from a uniform distribution $\mathcal {U}(0,10)$, yielding 10000 pairs of parameters. For each pair of parameters, we calculated the mean sum of rewards in the task over 500 simulated runs (thus 5M total simulations). Finally, we chose the pair of parameters that produced the largest mean sum of rewards as an approximation to the optimal parameters. Simulating the VLT with these parameters allows us to see whether the qualitative empirical effects—in particular any win-loss asymmetries—persist when using the best possible parameters for the learning algorithm. The optimal parameter values also provide some insight into the nature of the task itself—what the task structure is demanding of the learner. The simple randomized grid search method also allows us to visualize the 2-D payoff surface (Fig. 1, described below).

### Main results

We simulated the VLT for all N = 191 participants in Lin et al., (2020) who exceeded a minimal learning threshold, defined as achieving at least 65% correct selection in the final block; we discuss the remaining poorly-performing participants below. The model was run 200 times for each participant and so the aggregated results represent a mean of 191×200 = 38,200 model runs.

**Fig. 2:**
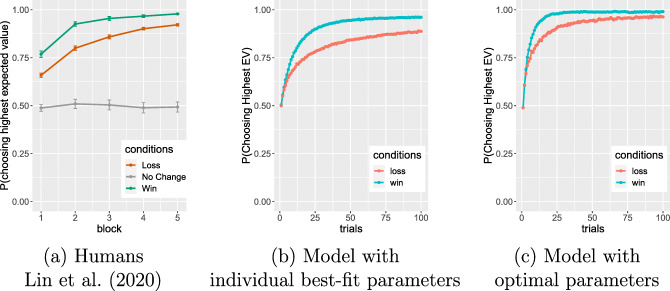
(a) Human participant results (*N* = 191) from Lin et al. (2020): Mean probability of selecting the correct stimulus from win pairs and loss pairs, across the 5 blocks (100 trials total), showing better performance for win pairs than loss pairs. Error bars represent one standard error (SE). (b) Model simulation of probability of correct selection for the 191 participants using best-fitting parameters for individuals. (c) Model simulation of probability of correct selection using optimal parameters; the asymmetry persists in this model, though it is quantitatively diminished

Figure 2b shows the aggregate results of the model simulating the 191 participants. The results are very similar to the empirical results (Fig. 2a), and in particular, there is a clear asymmetry in performance on the win and loss stimuli: the win pairs are learned better than the loss pairs. This difference diminishes with learning but persists through the final block.

We also simulated learning with optimal parameters—the settings of *α* and *β* that maximize expected reward. Figure 2c, shows the performance averaged over 5000 runs of the optimal parameter setting. The payoff surface is shown in Fig. 1, which plots the total expected reward earned in the task given different values of *α* and *β*. The optimal values are *α*^∗^ = 0.27,*β*^∗^ = 9.02, indicating that the best strategy is to update value estimates aggressively from recent past trials and accordingly exploit the learned better option in each condition. This is the result of the structure and setup of the task, and is a function of the probabilities (0.8 and 0.2) and number of trials. Probabilities closer to 0.5 (say 0.65 and 0.35) would impose a more difficult learning task and result in lower optimal *α* levels.

Even at optimal learning and exploration rates, the simulation results show that win trials are learned better than loss trials, though the asymmetry is quantitatively diminished. This suggests that the explanation of the win-loss asymmetry cannot be simply that participants have adopted suboptimal learning or exploration rates.

### Simulation of three other VLT studies

In this section we show that the learning asymmetry exhibited by the model of the VLT in Lin et al., (2020) also occurs when simulating three other studies that use the same general paradigm, but with different numbers of stimuli pairs and number of trials (Raymond and O’Brien, 2009; Rothkirch, Tonn, Köler, & Sterzer, 2017; Painter et al., 2014). Because we did not have access to individual participant data from these studies, we simulated the tasks using approximately optimal settings for *α* and *β*, using the method described above for find the optimal parameters.

**Fig. 3:**
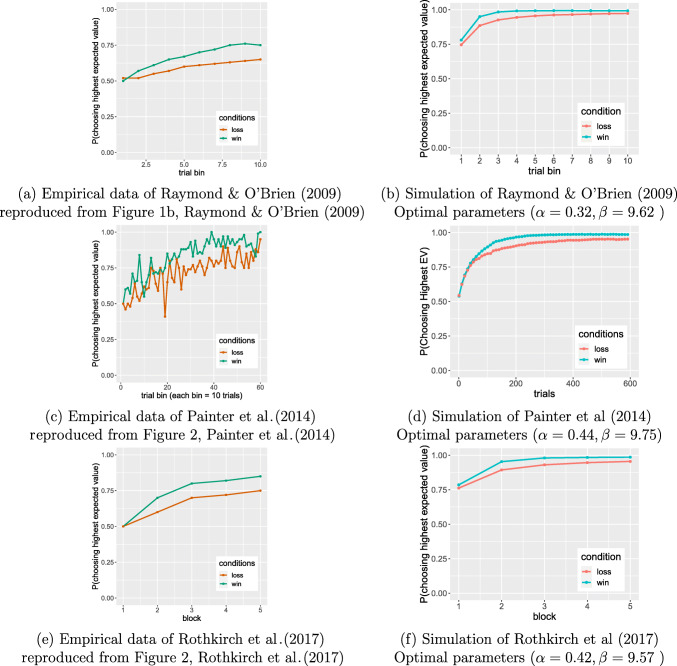
Simulations of three studies using the VLT. The difference in learning in wins and losses persists in these studies although they have different pairs stimuli or numbers of trials from Lin et al., (2020)

In Raymond and O‘Brien (2009) the VLT consisted of of six pairs of faces: two win pairs, two loss pairs, and two control pairs. Each pair was presented 100 times randomly in each block for a total of 6 blocks, yielding a total of 600 trials. On each trial, a choice led to a monetary outcome for win and loss trials (5 pence) with a probability of either 0.8 or 0.2 (Raymond & O’Brien, 2009). We simulated the probability of correct choice (mean of 10000 runs with optimal parameters: *α* = 0.32*β* = 9.62) within ten 10-trial bins to match the data display in Raymond and O’Brien (2009) (Fig. 3(a) and (b)). Both model and human participants show the win-loss asymmetry, though the model’s performance with optimal parameters is much higher than the humans.

Painter et al., (2014) used twelve pairs of flute glasses as stimuli, six of which were win pairs and the other six were loss pairs. Each pair was presented 10 times in each block for a total 5 blocks, yielding a total of 600 trials. The monetary outcome for win and loss trials (20 cents in AUD) occurred with a probability of either 0.8 or 0.2. During the last block, participants no longer received any feedback, indicating that participants only learned during the first 4 task blocks and were tested for their learning during the last block (Painter et al., 2014). The empirical results and optimal-parameter model simulation (mean of 10000 runs) is shown in Fig. 3(c) and (d). Again, both the empirical and simulated results show that the win condition was learned better than the loss condition, and in this experiment the human participants are much closer to the performance of the optimal model.

The final study that we simulated was Rothkirch et al., (2017). Their task consisted of four pairs of stimuli, two of which were win pairs and the remaining two were loss pairs. Each pair was presented 10 times in each block for a total of 5 blocks, yielding a total of 200 trials. The monetary outcome for win and loss trials (5 cents) occurred with a probability of either 0.8 or 0.2 (Rothkirch et al., 2017). The empirical results and optimal parameter model simulation (mean of 10000 runs) are shown in Fig. 3(e)^1^ and (f). There were no differences in participants’ learning of the two pairs of stimuli *within* the win condition (called “Reward” in Rothkirch et al., (2017)) and the loss condition (called “Punishment”). But again, both the empirical and simulated results show win pairs were learned better than loss pairs over the blocks.

## Explaining the win-loss asymmetry

The VLT paradigm in Lin et al., (2020) and the three experiments above each have seemingly symmetric payoff structures^2^ (Table 1). But our model predicts that asymmetric learning of wins and losses will occur across all the experiments. What gives rise to the asymmetry?

**Fig. 4:**
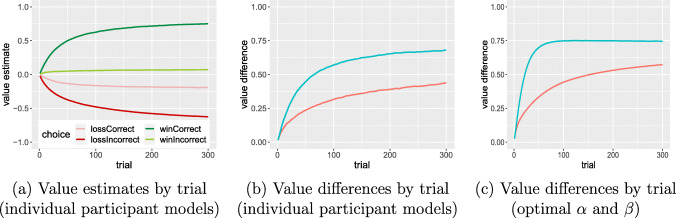
(a) Evolving mean value estimates for the four stimuli in win and loss pairs; mean computed from 30 runs of each of the 191 individual participant models. (b) Evolving mean differences in value estimates for win and loss conditions from the 191 individual participant models. (c) Evolving mean differences from the model with optimal *α* and *β* (5000 runs)

An examination of the evolving value estimates in the model reveals that they exhibit a different pattern for win and loss pairs over the course of the simulated experiment. The mean trial-by-trial value estimates for all choices in win and loss conditions for the 191 models with *α* and *β* fit to individual participants is shown in Fig. 4a, and b shows the corresponding *differences* in values between stimuli in the win and loss pairs. These differences are key because they are monotonically related to differences in probability of choice for each option. It is clear that the stimuli in the win pair are more sharply discriminated than the stimuli in the loss pair.

Why is this the case? In the win condition, the value estimates for the win-correct option approach the true expected value of 0.8 within the first 150 trials; this is not surprising because the win-correct option is sampled frequently. The win-incorrect option has still not approached the true expected value of 0.2 by the end of the experiment because it is sampled much less frequently and the initial estimate of zero still has its influence. Similarly, the loss-incorrect option is more slowly approaching the true expected value of − 0.8 because it is sampled less frequently than the loss-correct option, which is approaching the true expected value of − 0.2. But the result is that the value estimates of the loss pair stimuli are closer together, leading to comparatively greater choices of the incorrect loss option than the incorrect win option; put differently, model choices in the loss pair are noisier. The asymmetry persists when *α* and *β* are set to their optimal values (Fig. 4c). In short, throughout the task, the loss stimuli remain more poorly discriminated than win stimuli.

## Individual differences in the VLT

We have shown that a simple RL error-driven learning model provides an explanation of win-loss learning asymmetries in the superficially symmetric VLT paradigm, and have shown that this asymmetry persists whether the learning and exploration parameters are set to maximize empirical fit to individual participants, or are set to the computationally rational optimal setting to maximize task reward. We now examine the extent to which the asymmetry persists for all participants, and whether variation in the model’s learning parameters can account for individual differences.

Empirical data from Lin et al., (2020) suggest that the striking asymmetric learning pattern does not characterize all individuals: a subset of participants learned both conditions nearly equally well. To help visualize this participant variation, we characterized the learning asymmetry for each participant (in the N = 191 who achieved at least 65% correct selection in the last block) by computing the difference between mean probabilities of correct selection of win and loss stimuli across the 5 blocks. We then separated participants into two groups using a median split on this difference measure. We refer to the group with lower win-loss differences as the *Nearly Equal Learner Group*, and the group with greater win-loss differences as the *Unequal Learner Group*.

**Fig. 5:**
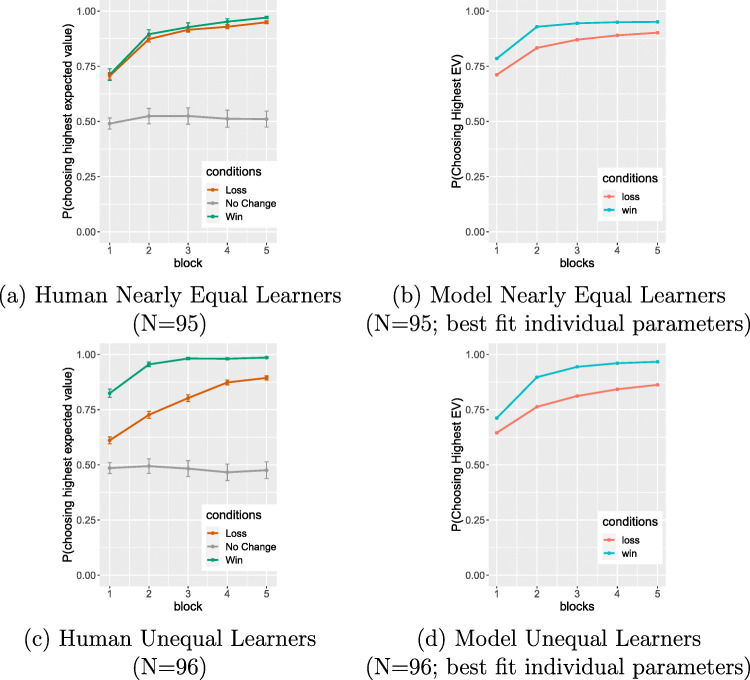
Human data (error bars represent one SE.) and model simulations for two groups of participants created by a median split on the learning asymmetry; see text for details

Figure 5 shows the empirical (left panel) and best-parameter-fit model-simulation (right panel) learning curves for the Nearly Equal Learners (top row) and Unequal Learners (bottom row). Note that these model simulations are identical to the ones presented above for the N = 191 participants in Lin et al., (2020);^3^ we are simply splitting those results into the two different groups. The key result here is that the asymmetry is diminished in the simulation of the Nearly Equal Learners, though not to the extent observed in the empirical means. This suggest that variation in *α* and *β* provides a partial account of the individual variation in the win-loss asymmetry.

**Fig. 6:**
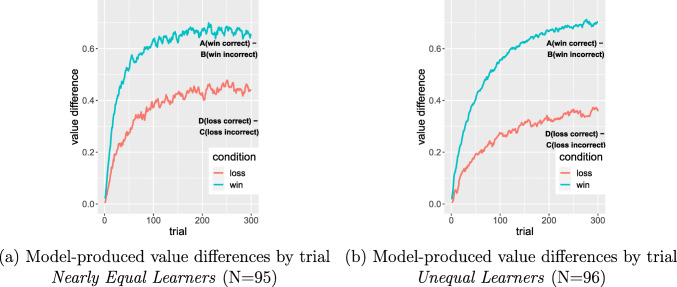
(a) Evolving mean differences in value estimates for win and loss conditions from the 95 models of the Nearly Equal Learner participants. (b) Evolving mean differences in value estimates for win and loss conditions from the 96 models of Unequal Learners

We also explored the effect of the individual parameter variation on predicted estimated values for participants in the two groups (Fig. 6). Consistent with the analysis presented above, the mean differences in value estimates for win and loss conditions from models of participants in the Nearly Equal Learner group are smaller than those from the models of participants in the Unequal Learners’ group.

Each individual participant also differed in the specific experiences on each trial. Although it seems unlikely that these experience differences could account for the individual differences we observed, we also ran simulations of the model using the actual experience of each individual participant—that is, forcing the model to experience the exact same trial conditions in the same order as the participants. We then computed optimal learning parameters for these individual experiences to assess whether experience alone might lead to upper bounds on performance that vary enough to account for some of the observed performance differences. We did not observe any differences in the optimal model simulations, suggesting that random experience differences cannot account for the observed variation in individual performance (Simulation results in the Supplementary Materials).

Finally, the model simulates the performance of most participants who did not achieve the 65% correct selection threshold (some of whom were operating nearly at chance). Setting either *α* or *β* to very low levels yields poor performance. It is possible to further divide the poor performing participants into subgroups who learned neither win or loss associations (N = 23), or who learned wins slightly better than losses (N = 17), or losses slightly better than wins (N = 8). Only the latter small group of participants (8 of 287) cannot be accounted for by the model. Simulation results of these four subgroups are in the Supplementary Materials).

**Fig. 7:**
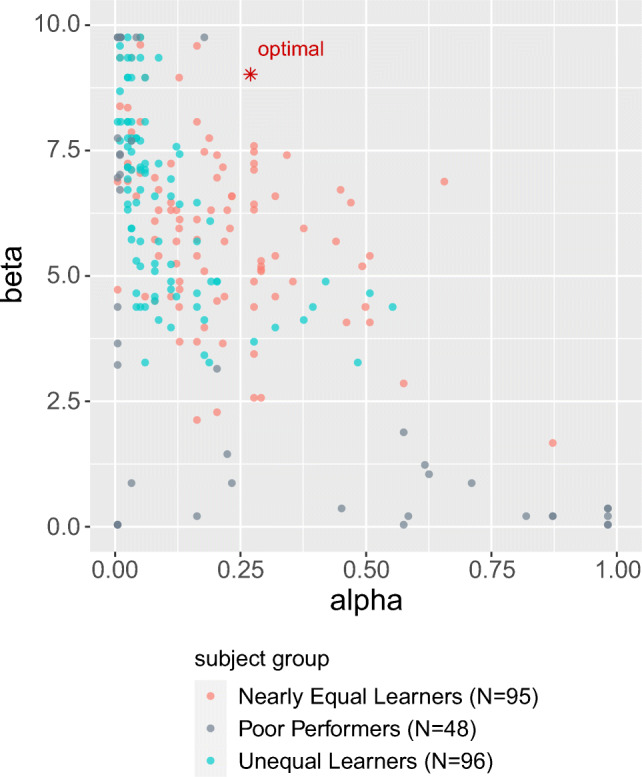
Individual best-fit parameters for all participants, distinguishing the three groups: *Nearly Equal Learners*, *Unequal Learners*, and *Poor Performers*. Parameter settings for the *Nearly Equal Learners* are closer to the optimal setting (see Fig. 1); the model thus predicts that these participants will have higher overall performance, a prediction that is consistent with the data

Figure 7 shows the best-fitting *α* and *β* parameters for each of the 287 participants, color coded for each of the three groups: *Unequal Learners*, *Nearly Equal Learners*, and *Poor Performers*. What is clear from this plot is that the Nearly Equal Learners have parameter values closer to the optimal parameters. The model thus predicts that these participants will have the highest overall performance, a prediction that is confirmed empirically (See Supplemental Materials).

## Modeling a learning outcome memory task

Following the VLT, Lin et al., (2020) administered a post-learning memory task that aims to probe participants’ explicit knowledge of the outcome associated with each stimulus (scenes) that appeared in the VLT.^4^ The task included the 6 VLT scenes and 12 new scenes. VLT scenes were presented 4 times each and 12 new images each appeared twice. Participants indicated the outcome associated with each image as follows: 1) *very likely to win*, 2) *occasionally win*, 3) *no change*, 4) *occasionally lose*, 5) *very likely to lose*, 6) *none* (indicating a new image).

**Fig. 8:**
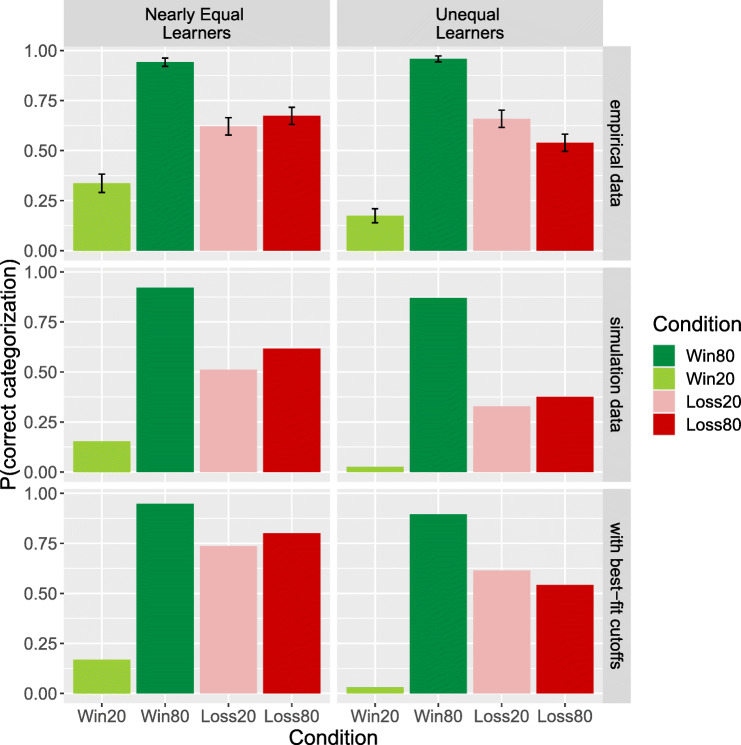
Memory task results (top panels) from human Nearly Equal Learners (N = 95) and Unequal learners (N = 96) and categorization of stimuli given simulated value estimates for the two groups of participants (error bars represent one SE). Simulation data (second-row panels) show the mean probability of correct categorization for the two groups based on 1000 sets of plausible cutoffs. Simulation with best-fit cutoffs (third-row panels) shows the probability of correct categorization for the two groups based on the set of cutoffs that fit empirical data the best. The best-fit set (1: *very likely win* (value estimates > + 0.51), 2: *occasional win* (value estimates > + 0.17), 3: *no change* (value estimates > − 0.17), 4: *occasional loss* (value estimates > − 0.51), and 5: *very likely loss* (value estimates ≤− 0.51)) are decided by the minimum mean squared error between P(correct categorization) from simulation and empirical data

Figure 8, top panel, shows the human results for the two groups of participants (Nearly Equal Learners and Unequal Learners). In both groups, there is a clear interaction: the Win-80 stimulus was very accurately categorized but the Win-20 stimulus was categorized poorly. Each of the two Loss stimuli were categorized about equally well, better than Win-20 but not as accurately as Win-80. In short, there is a clear valence difference but also an interesting interaction. And given this interaction, when collapsing across the paired stimuli, accuracy on the Loss stimuli is slightly overall higher than Win stimuli—a counter-intuitive result given the choice performance asymmetry.

We extended the learning model to also provide an account of the performance on the memory probe task, for those stimuli that were part of the VLT. The simple hypothesis we pursued is the following: participants would make the categorical judgments based on their learned values estimates for each stimuli, using a set of reasonable thresholds over these estimates to yield the five categories.

In our initial exploration, we hand-picked the following intuitively reasonable ranges for the thresholds or breakpoints for mapping values estimates into the four categories (we also found empirical best-fit thresholds, described below). Recall that the observed rewards for the model were + 1, − 1, or 0.

- *very likely win* was defined as value estimates ≥ highest threshold, where highest threshold ∈ [+ 0.50,+ 0.70];
- *occasional win* was defined as value estimates ≥ high threshold, where high threshold ∈ [+ 0.17,+ 0.23];
- *no change* was defined as value estimates ≥ low threshold, where low threshold ∈ [− 0.23,− 0.17];
- *occasional loss* was defined as value estimates ≥ lowest threshold, where lowest threshold ∈ [− 0.70,− 0.50]; and
- *very likely loss* was defined as value estimates ≤ lowest threshold

Note that when these thresholds are applied to the *true* values of stimuli, they yield the intuitively correct categorizations of stimuli that were used as the definition of the correct responses for computing the empirical accuracy scores reported in Lin et al., (2020).

We then took the value estimates for win and loss stimuli from the models for each of the 191 participants who reached our previously specified learning criterion and sampled 1000 sets of thresholds from their plausible ranges to create simulated responses to the memory task.

Figure 8, second row, shows the probability of categorizing stimuli correctly based on the cutoffs above for the two groups of simulated value estimates. Figure 8, third row shows the results with cutoff thresholds chosen to maximize empirical fit (minimize mean-squared error between predicted and observed accuracies). It is clear that the modeling results recover the key qualitative patterns in the human data. From the 1000 sets of sampled thresholds, when we set the cutoffs to maximize the categorization accuracies, we found the same results as when the cutoffs maximize empirical fit (see Fig. 8).

## Discussion

The value learning task (VLT) developed by Raymond and O’Brien (2009) is a simple and popular paradigm for studying value learning and the effects that learned value have on subsequent processing of valued stimuli. The VLT paradigm involves learning and choice in an uncertain environment, and that uncertainty imposes the classic exlporation-exploitation tradeoff on the learner. But the standard paradigm, despite the apparent symmetry in payoff structure, yields a contrast between wins and losses: choice performance on win stimuli is better than loss stimuli (Lin et al., 2020; Rothkirch et al., 2017), and this pattern holds whether participants receive points or monetary rewards, and even when they are explicitly instructed about the structure of the task.

We developed a simple model of the VLT based on a standard error-driven learning rule, soft-max choice (to balance exploration and exploitation), and neutral (zero) initial value estimates. This model produces the asymmetry in learning gains and losses that is evident in human performance. This is the case despite (a) the task itself having a symmetric design; (b) the learning and choice rules having no special role for valence; and (c) allowing the learning and choice rule parameters to vary widely and include optimal settings for the task. The model furthermore yields an explanation: the asymmetric learning pattern arises from an interaction of incremental learning, exploitation while exploring, and neutral initial value estimates. As a consequence the learned values of the loss stimuli are discriminated less well than the win stimuli. We have shown this asymmetric learning pattern arises in three other experimental tasks that have a very similar structure to the VLT in Lin et al., (2020).

Nevertheless, this asymmetric learning pattern for win and loss stimuli does not arise uniformly across participants: a subset of the participants learn wins and losses nearly equally well. The model partially explains this variation in terms of individual variation in the learning and exploration parameters. From simulating each participant’s individual task experience with both the optimal parameters and best-fitting parameters, we were also able to rule out random variations in task experience as the source of the individual differences.

A simple extension of the model that uses the learned value estimates to simulate a post-learning outcome memory task provides further evidence for the asymmetric value estimates that the model naturally produces, and thus indirectly for our assumption of an initial neutral value estimate. It yields the observed win-loss interaction in the human data and even accounts for the surprising finding that accuracy in categorizing outcomes of loss-stimuli is slightly better than win-stimuli (Results of overall correct categorization for win- and loss-stimuli in Supplementary Materials). However, despite nearly equal or unequal learning of the win- and loss-stimuli in the behavioral task, the qualitative effects on subsequent memory performance were the same. This suggests that the win-loss asymmetry in learning does not directly drive effects on subsequent tasks. Instead, the learned value estimates were better predictors of the subsequent memory task. Thus, a promising avenue for future work is to quantitatively model value learning as we have done here, and use the learned value estimates as parameters of computational models of downstream tasks.

There are several aspects to note for this model. First, we have selected a standard softmax rule as the model’s choice rule. Other choice rules such as the reinforcement learning diffusion decision (Fontanesi, Gluth, Spektor, & Rieskamp, 2019) and *𝜖*-softmax (Shteingart, Neiman, & Loewenstein, 2013) may also be able to predict the empirically observed learning asymmetry. While the standard softmax rule associates action selection with the estimated action values and indicate that action selection can become sensitive to the action values, the *𝜖*-softmax choice rule (Shteingart et al., 2013) can associate values and choices while maintaining a certain level exploration. This could potentially reduce sampling bias in the simulations, which partially contributes to the learning asymmetry. Similarly, an adaptive inverse temperature parameter that reduces during the task may also reduce the sampling bias by maintaining exploration. Besides different choice rules, future work may also explore how other learning rules such as the decay learning rules (Don, Otto, Cornwall, Davis, & Worthy, 2019) may affect the model prediction on the win and loss learning asymmetries.

Second, this model also provides explanations for the win-loss asymmetry with minimal assumptions where two parameters, the learning rate (*α*) and the inverse temperature parameter (*β*), are considered. Other models may differentiate between win and loss contexts and implement different learning rates correspondingly (Palminteri et al., 2015). Such models are worth exploring for future work.

In addition, it is worth noting that, for this model, valence is relevant insofar as exploitation wants to pursue greater reward. But valence in the sense of positive/negative does not play a special role. Therefore, such a computational model is very useful for researchers to have as a baseline model for any value learning task, to draw out the implications of the simplest set of assumptions that do not assume a special role for positive/negative valence. In this sense, it is also a way to put into sharper focus any real valence-related differences that do emerge.

We discuss two limitations of our model. First, although the model simulations reflect the general characteristics of human performances by people in different groups, the model cannot account for the performance of the small percentage (< 3*%*) of individuals who performed better in the loss condition than the win condition. It is possible that individuals who learned losses better than wins have a different internal reward function that transforms point or monetary observations into an internal reward signal, but this could be very challenging to estimate. Second, our model does not take into account the possibility that humans may also learn the *structure* of the task in ways that allow them to update value estimates for the stimulus in the pair *other* than the one that is chosen. In other words, in the VLT, feedback on one stimulus in a pair does provide information about the value of the other stimulus. It is possible that this more efficient task structure learning accounts for some of the performance differences of participants in the Nearly Equal Learners group. A more sophisticated structured Bayesian RL model could be developed to account for such learning.

The model-based analysis provides some insights into what we could do to reduce the asymmetry in learning in the VLT, without compromising the task’s symmetric design. Again, the asymmetric pattern is a result of the interaction of incremental learning, the balance between exploration and exploitation, and zero initial values. One clear way to reduce the asymmetry in learning is to adjust the initial values for the actions by allowing an extra block at the beginning of the experiment as a purely exploration phase, where participants are instructed to learn as much about each option as possible, without concern for exploitation. Adjusted initial values could lead to a smaller difference in learned value estimates between win and loss conditions, and may subsequently produce smaller differences in performance for categorizing win and loss stimuli. This solution needs to be tested with further empirical work.

## Electronic supplementary material

Below is the link to the electronic supplementary material.

(PDF 215 KB)

## Footnotes

1 In Fig. 3e Win and loss correct rates are calculated as the mean correct rates of patient group and healthy control groups.

2 It is worth noting that there is more than one way to define the *symmetry* of the task. Our notion of symmetry is based on the symmetric payoff structure of the task. However, *symmetry* could also be represented by 1) equal absolute expected values (EVs) of the correct win and loss options and equal differences between the correct and incorrect options in win and loss conditions; 2) equal absolute EVs of the incorrect win and loss options and equal differences between the correct and incorrect options in win and loss conditions. Simulations of the latter two task structures with the optimal parameters of this VLT task show that the win-loss asymmetry persists (see Supplementary Materials for results).

3 This individual difference analysis was not included in Lin et al., (2020)

4 Lin et al., (2020) reported memory task performance for all participants who met learning criteria (N = 191).

## References

[CR1] Aberg, K., Müller, J., & Schwartz, S. (2017). Trial-by-trial modulation of associative memory formation by reward prediction error and reward anticipation as revealed by a biologically plausible computational model. *Frontiers in Human Neuroscience, 11*. 10.3389/fnhum.2017.00056. 10.3389/fnhum.2017.00056 PMC5309218 28261071

[CR2] Brosch T, Sander D (2013). Neurocognitive mechanisms underlying value-based decision-making: from core values to economic value. Frontiers in Human Neuroscience.

[CR3] Daw, N. (2011). Trial-by-trial data analysis using computational models. In *Decision making, affect, and learning: attention and performance XXIII*. 10.1093/acprof:oso/9780199600434.003.000110.1093/acprof:oso/9780199600434.003.0001: Oxford University Press.

[CR4] Daw N, Niv Y, Dayan P (2005). Uncertainty-based competition between prefrontal and dorsolateral striatal systems for behavioral control. Nature Neuroscience.

[CR5] Della LC, Chelazzi L (2009). Learning to attend and to ignore is a matter of gains and losses. Psychological Science.

[CR6] Don HJ, Otto AR, Cornwall AC, Davis T, Worthy DA (2019). Learning reward frequency over reward probability: A tale of two learning rules. Cognition.

[CR7] Fontanesi L, Gluth S, Spektor MS, Rieskamp J (2019). A reinforcement learning diffusion decision model for value-based decisions. Psychonomic Bulletin & Review.

[CR8] Gershman SJ, Daw ND (2017). Reinforcement learning and episodic memory in humans and animals: An integrative framework. Annual Review of Psychology.

[CR9] Kahneman D (2003). Maps of bounded rationality: Psychology for behavioral economics. American Economic Review.

[CR10] Lewis, R., Howes, A., & Singh, S. (2014). Computational rationality: Linking mechanism and behavior through bounded utility maximization. *Topics in Cognitive Science, 6*. 10.1111/tops.12086. 10.1111/tops.12086 24648415

[CR11] Lin Z, Cabrera-Haro LE, Reuter-Lorenz PA (2020). Asymmetrical learning and memory for acquired gain versus loss associations. Cognition.

[CR12] Maia TV (2010). Two-factor theory, the actor-critic model, and conditioned avoidance. Learning & Behavior.

[CR13] Montague PR, Hyman SE, Cohen JD (2004). Computational roles for dopamine in behavioural control. Nature.

[CR14] Moutoussis M, Bentall RP, Williams J, Dayan P (2008). A temporal difference account of avoidance learning. Network: Computation in Neural Systems.

[CR15] Mowrer, O. (1960). Learning theory and behavior.

[CR16] Painter DR, Kritikos A, Raymond JE (2014). Value learning modulates goal-directed actions. The Quarterly. Journal of Experimental Psychology.

[CR17] Palminteri S, Khamassi M, Joffily M, Coricelli G (2015). Contextual modulation of value signals in reward and punishment learning. Nature Communications.

[CR18] Palminteri S, Lebreton M (2021). Context-dependent outcome encoding in human reinforcement learning. Current Opinion in Behavioral Sciences.

[CR19] Rangel A, Camerer C, Montague PR (2008). A framework for studying the neurobiology of value-based decision making. Nature Reviews Neuroscience.

[CR20] Raymond JE, O’Brien JL (2009). Selective visual attention and motivation: The consequences of value learning in an attentional blink task. Psychological Science.

[CR21] Rothkirch M, Tonn J, Köler SJ, Sterzer P (2017). Neural mechanisms of reinforcement learning in unmedicated patients with major depressive disorder. Brain: A Journal of Neurology.

[CR22] Savage, L.J. (1972). *The foundations of statistics*. Courier Corporation.

[CR23] Shteingart H, Neiman T, Loewenstein Y (2013). The role of first impression in operant learning. Journal of Experimental Psychology: General.

[CR24] Singh S, Lewis RL, Barto AG, Sorg J (2010). Intrinsically motivated reinforcement learning: An evolutionary perspective. IEEE Transactions on Autonomous Mental Development.

[CR25] Sutton RS, Barto AG (2018). Reinforcement learning: an introduction.

