# Supplementary material for: Why do valence asymmetries emerge in value learning? A reinforcement learning account
Source: Cogn Affect Behav Neurosci. 2022 Dec 28;23(3):543–56. doi: 10.3758/s13415-022-01050-8 (PMC10390629; doi:10.3758/s13415-022-01050-8)
Supplement: Supplementary file 1 — (PDF 215 KB) [file 13415_2022_1050_MOESM1_ESM.pdf]

Supplemental Materials

Table 1: The payoff structures that lead to 1) equal absolute EVs of the correct win and loss options and equal differences between the correct and incorrect options in win and loss conditions; 2) equal absolute EVs of the incorrect win and loss options and equal differences between the correct and incorrect options in win and loss conditions. All rewards are standardized.

Condition	Stimulus	Outcomes and Probabilities	Expected Value
Structure 1:			
Win pair	A	+1 (p = 0.6), 0 (p = 0.4)	0.6
Win pair	B	0 (p = 0.6), +1 (p = 0.4)	0.4
Loss pair	C	-1 (p = 0.6), 0 (p = 0.4)	-0.6
Loss pair	D	-1 (p = 0.8), 0 (p = 0.2)	-0.8
Structure 2:			
Win pair	A	+1 (p = 0.6), 0 (p = 0.4)	0.6
Win pair	B	0 (p = 0.6), +1 (p = 0.4)	0.4
Loss pair	C	-1 (p = 0.2), 0 (p = 0.8)	-0.2
Loss pair	D	-1 (p = 0.4), 0 (p = 0.6)	-0.4

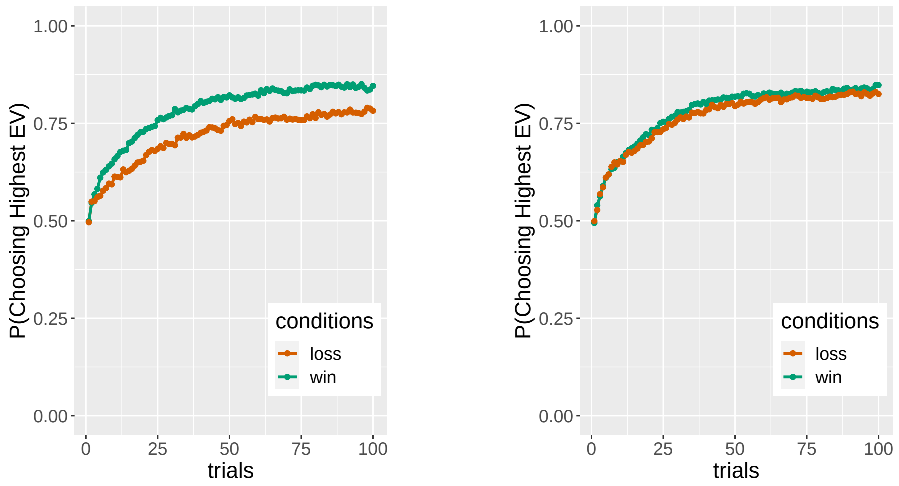

(a) Model, with payoff *Structure 1* (b) Model, with payoff Structure 2

Figure 1: Model simulations (5000 runs for each participant) of the VLT by different payoff structures from Table 1 — using optimal parameters. With *Structure 2*, when the EVs of the incorrect win and loss options are the same, the win-loss asymmetry is decreased. In this case, the two win options have EVs farther away from the initial value of zero. This result is consistent with the influence of the initial values in our explanation for the learning asymmetry.

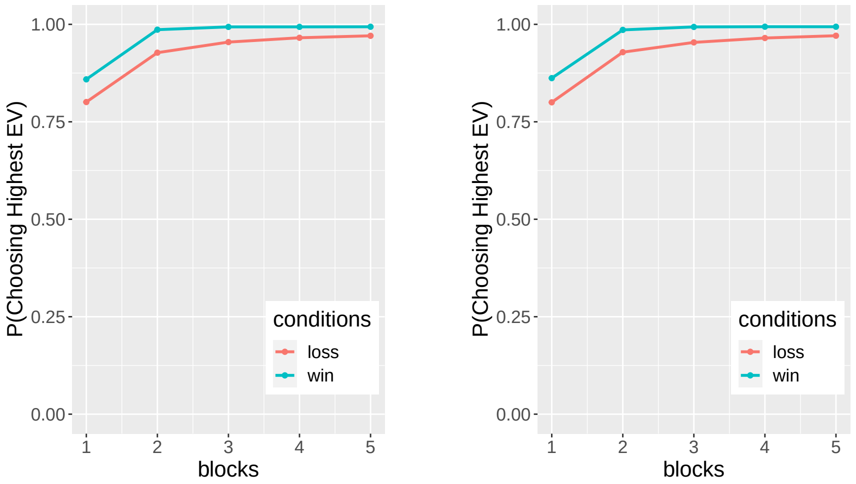

(a) Model, *Nearly Equal Learners* (N = 95; optimal parameters) (b) Model, *Unequal Learners* (N = 96; optimal parameters)

Figure 2: Model simulations (200 runs for each participant) of the VLT with the exact experiences of human participants — using optimal parameters.

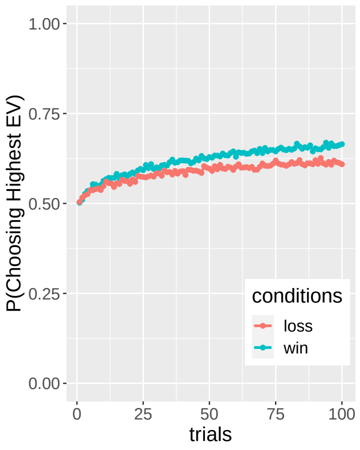

(a) Model,
all *Poor Performers* (N=48)

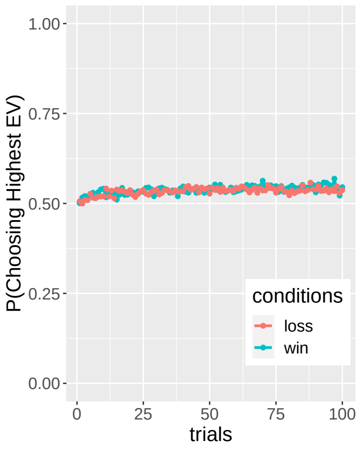

(b) Model,
Poor Performers, ”neither” group (N=23)

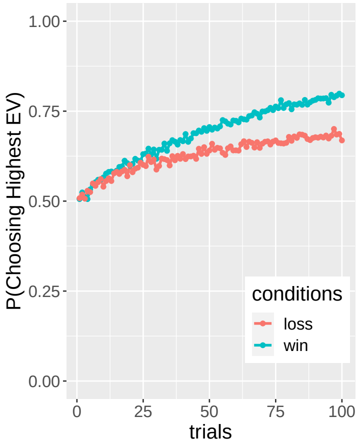

(c) Model,
Poor Performers, ”win-only” group (N=17)

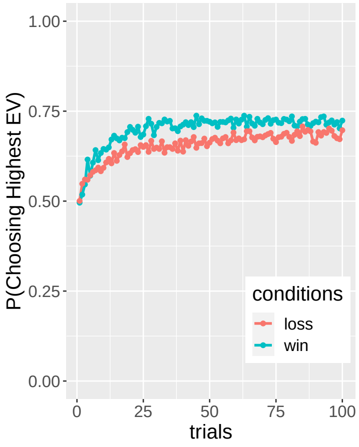

(d) Model,
Poor Performers, ”loss-only” group (N=8)

Figure 3: (a). Model simulation for *Poor Performers* (each=200 simulations). (b). Model simulation for *Poor Performers* who learned neither stimuli. The correct selection rates stay around 50% for both stimuli. (c) & (d). Model simulations for *Poor Performers* who learned only win stimuli and those who learned only loss stimuli. The simulations do not reflect when loss stimuli are learned better win stimuli.

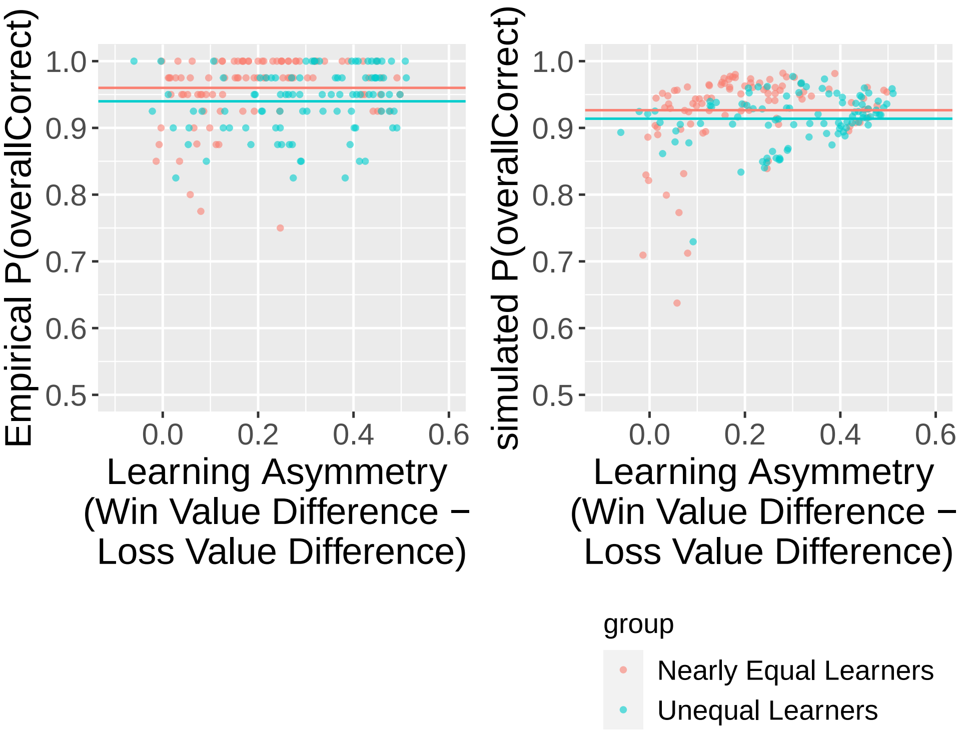

Figure 4: Learning asymmetry and overall performances by human subjects (left; N = 191) and model simulation with best-fit parameters (right). Vertical lines indicate the means of overall performance by each subject group. Learning asymmetry is given by the *differences between the value differences of win stimuli and the value differences of loss stimuli*: larger absolute differences indicates larger asymmetry between wins and losses. Overall performance is the mean P(correct) of wins and losses in the last block. Our model predictions are consistent with empirical data: *Nearly Equal Learners* generally have lower learning asymmetry and higher overall performance.

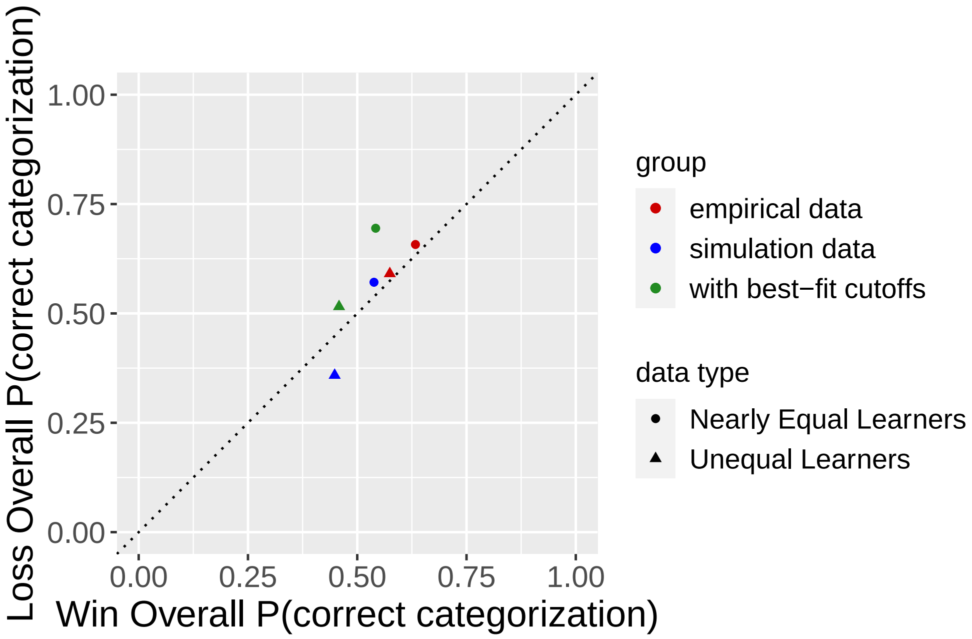

Figure 5: Overall correct categorization for win- and loss-stimuli by *Nearly Equal Learners* and *Unequal Learners* in empirical data, simulated data, and simulations with best-fit cutoffs. Our simulated results show the observed win-loss interaction in the human data, including the surprising finding that accuracy in categorizing outcomes of loss-stimuli is slightly better than win-stimuli. This finding does not appear in results simulated with sampled cutoffs, but it appears in results simulated with best-fit cutoffs.
